# Supplementary material for: Prognostic value of SPARC in hepatocellular carcinoma: A systematic review and meta-analysis
Source: PLoS One. 2022 Aug 18;17(8):e0273317. doi: 10.1371/journal.pone.0273317 (PMC9387809; doi:10.1371/journal.pone.0273317)
Supplement: Attachment — Submitted filename: Response to Reviewers.docx [file pone.0273317.s002.docx]

Dear Dr Raj Kumar Koiri

*PLoS One*

Thank you very much for giving us an opportunity to resubmit the enclosed manuscript entitled “Prognostic value of SPARC in hepatocellular carcinoma: a systematic review and meta-analysis” (PONE-D-21-33504) for possible publication in *PLoS One*.

On behalf of my coauthors, I would like to thank you and the reviewers for your valuable suggestions and comments. I have carefully incorporated all comments of reviewers into the newly revised manuscript. All major changes are in red in this revised version.

My responses to the Reviewers’ comments are below. I believe all their concerns have been addressed, and the quality of the revised manuscript has been improved significantly. I sincerely hope the revised manuscript will be acceptable to you and the reviewers. If you have any further questions about the manuscript, please feel free to contact me. Your consideration for this manuscript to be published in *PLoS One* is much appreciated.

Kind regards,

Yunhong Xia

Department of Oncology, the Fourth Affiliated Hospital, Anhui Medical University, Hefei, Anhui, China

E-mail: yhxia21@sina.com

**Reviewer Comments to the Author**
Reviewer #1:

1. 1&2&3. What are the justifications for the exclusion criteria? For instance, why were similar studies excluded, how was their ‘similarity’ measured, and how did the authors decide which study among similar studies to include?

**Response:** Thank you very much for your constructive and helpful suggestions. In this paper, similarity refers to different articles using the same case. In order to eliminate misunderstandings, we corrected the manuscript again. Please see Line 106-119.

2. The ‘Cochrane handbook for systematic reviews of treatments’ (please cite this resource/include it in the references) was used to evaluate publication bias; however, the criteria is different from what is described in the handbook. For instance, what does ‘Other bias’ mean?

**Response**: Thank you very much. The wrong statement has been corrected. The right quality evaluation of the literature is as flowed: The quality of the included studies was appraised by two authors using the risk bias assessment technique in the Cochrane Handbook for Systematic Reviews of Treatments 5.1.0, which assesses the risk of publication bias. Investigators and outcome assessors were blinded to all information on random sequence generation and allocation concealment. All articles were evaluated for completeness of outcomes data and selective reporting of research outcomes. Please see Line 130-136.

3. The conclusions regarding overall survival and disease-free survival are supported by only 6 and 2 studies, respectively. Due to the small number of studies reviewed and as mentioned in the article, no comprehensive evidence is presented to support the conclusions.

**Response**: Thanks a lot. Although only six studies were included in terms of overall survival, data analysis showed that increased expression of SPARC was significantly associated with OS differences in HCC patients. Therefore, the expression of SPARC has an impact on HCC patients, but more high-quality studies are needed to confirm this outcome in the future. This belongs to the limitations of this meta-analysis. Please see Line 256-268.

4. A number of sections in this article contain grammatical errors, making it difficult to understand the text. Acronym definitions are not consistent in terms of letter case and whether the acronym is included in parenthesis or the other way around.
   The ‘Country’ column in Table 1 seems to be the author’s affiliation, not the country.

**Response**: Thank you very much for your helpful suggestions. We invited native English speakers to polish the language of this paper. In addition, Table 1 has been checked and the wrong words have been removed in revised version.

5. Would it be possible to include figures of higher quality? It’s difficult to read the blurred text in figures.

**Response**: Thank you for your valuable suggestions. All figures have been checked. The figures of higher quality have been added instead of the old figures. Please see the figure files.

Reviewer #2: The authors are presenting a novel meta-analysis to analyze the predictive significance of SPARC in Hepatocellular Carcinoma patients.
Regardless of the small sample size, the data presented was novel and can be utilized to build future research in trying to pinpoint the association between SPARC and HCC
I do not have any comments for the authors.

**Response**: Thank you very much for your constructive and helpful suggestions.

Reviewer #3: The current manuscript presented a review and meta-analysis to evaluate the reported evidences in scientific domain in order to understand SPARC expression and the prognosis of patients with HCC. The attempt is worth trying but investigators left noticeable methodological flaws in conduct of this meta-analysis/reporting - making results less reliable. Some of Points are mentioned below:

1. Authors refer present work as Network Meta analysis which is incorrect. (L99)

**Response**: Thank you very much for your helpful suggestions. The word “network meta-analyses” has been modified to “meta-analyses”. Please see Line 88.

2. Authors have not provided Search Strategy Algorithms. The described search strategy in manuscript is of poor quality.

**Response**: Thank you very much for your constructive comments. The detailed search strategy has been added into the revised manuscript. Please see Line 90-105.

The retrieval strategy was as follows: (1) PubMed: (“Carcinoma, Hepatocellular”[Mesh] OR “Carcinoma, Hepatocellular”[All Fields] OR “HCC”[All Fields] OR “liver cancer”[All Fields]) AND (“Osteonectin”[Mesh] OR “Osteonectin”[All Fields] OR “SPARC”[All Fields] OR “Secreted protein acidic and cysteine rich”[All Fields]) AND (“Prognosis”[Mesh] OR “Prognosis”[All Fields] OR “Prognostic”[All Fields] OR “Survival Analysis”[Mesh] OR “Survival”); (2) Embase: (“hepatocellular carcinoma”: ti, ab, kw OR hcc: ti, ab, kw OR “liver cancer”: ti, ab, kw) AND (osteonectin OR sparc OR secreted) AND protein AND acidic AND cysteine AND rich AND (prognosis: ti, ab, kw OR prognostic: ti, ab, kw OR survival: ti, ab, kw); (3) Web of Science: TOPIC: (hepatocellular carcinoma OR HCC OR liver cancer) AND TOPIC: (Osteonectin OR SPARC OR Secreted protein acidic and cysteine rich) AND TOPIC: (Prognosis OR Prognostic OR Survival); (4) CNKI: keywords (hepatocellular carcinoma OR HCC OR liver cancer) and (Prognosis OR Prognostic OR Survival) in Chinese.
3. Systematic Review part of manuscript is incomplete, and there is no qualitative synthesis of evidence based on previously reported work.

**Response**: Thank you very much. Systematic Review part of manuscript has been modified (Line 149-155). The initial systematic search identified 1235 studies. After eliminating duplicates, 195 articles remained. Based on the title and abstract screening, 72 irrelevant articles were excluded, and 45 were further screened for assessment of eligibility. Thirty-nine studies that did not meet our inclusion criteria were excluded, leaving six for inclusion in the meta-analysis. Fig 1 shows the literature search and article selection processes.
4. Authors report that present work included the studies between 2019 and 2020 (L173), however, Table 1 does not comply with that criteria. The manuscript has many such contradictions- seems authors need to verify their data and description text.

**Response**: Thank you very much for your kind comments. The wrong word “2019” has been modified to “2009”. Please see Line 161.

5. Reason for the selection of Random Effects Model is insufficient. Results are insufficiently described which makes difficult to follow the conclusion and discussion part of manuscript.

**Response**: Thank you very much. In statistical analysis, the Q test was used to determine the size of I2 response heterogeneity: if P >0.1 and I^2^<50%, it indicates statistical homogeneity, and a fixed-effect model was used to conduct the meta-analysis; if P<0.1 or I^2^>50%, it indicates statistical heterogeneity, and a random effect model was used to conduct the meta-analysis and subgroup analysis to investigate the source of heterogeneity [1, 2]. Therefore, in this study, a randomized controlled model was selected due to I^2^>50%. Meanwhile, the confusing sentence has been removed in revised version and the right reason has been added into the revised manuscript. Please see Line 190-191.

References

1.	Sohouli MH, Fatahi S, Lari A, Lotfi M, Seifishahpar M, Gaman MA, et al. The effect of paleolithic diet on glucose metabolism and lipid profile among patients with metabolic disorders: a systematic review and meta-analysis of randomized controlled trials. Crit Rev Food Sci Nutr. 2021:1-12. Epub 2021/01/26. doi: 10.1080/10408398.2021.1876625. PubMed PMID: 33492173.

2.	Schwingshackl L, Zahringer J, Nitschke K, Torbahn G, Lohner S, Kuhn T, et al. Impact of intermittent energy restriction on anthropometric outcomes and intermediate disease markers in patients with overweight and obesity: systematic review and meta-analyses. Crit Rev Food Sci Nutr. 2021;61(8):1293-304. Epub 2020/05/05. doi: 10.1080/10408398.2020.1757616. PubMed PMID: 32363896.
