# Supplementary material for: Prognostic value of SPARC in hepatocellular carcinoma: A systematic review and meta-analysis
Source: PLoS One. 2022 Aug 18;17(8):e0273317. doi: 10.1371/journal.pone.0273317 (PMC9387809; doi:10.1371/journal.pone.0273317)
Supplement: Attachment — Submitted filename: Response to Reviewers.docx [file pone.0273317.s003.docx]

Dear Dr Raj Kumar Koiri,

*Plos One*

Thank you very much for giving us an opportunity to resubmit the enclosed manuscript entitled “**Prognostic value of SPARC in hepatocellular carcinoma: a systematic review and meta-analysis**” (PONE-D-21-33504R1) for possible publication in *Plos One*.

On behalf of my coauthors, I would like to thank you and the reviewers for your valuable suggestions and comments. I have carefully incorporated all comments of reviewers into the newly revised manuscript. All major changes are in **red** in this revised version.

My responses to the Reviewers’ comments are below. I believe all their concerns have been addressed, and the quality of the revised manuscript has been improved significantly. I sincerely hope the revised manuscript will be acceptable to you and the reviewers. If you have any further questions about the manuscript, please feel free to contact me. Your consideration for this manuscript to be published in *Plos One* is much appreciated.

Best regard,

Yunhong Xia

Department of Oncology, the Fourth Affiliated Hospital, Anhui Medical University, Hefei, Anhui, China

Email: [yhxia21@sina.com](mailto:yhxia21@sina.com)

Reviewer #4: In the manuscript titled “Prognostic value of SPARC in hepatocellular carcinoma: a systematic review and meta-analysis” the authors, with the help of data mining evaluated the SPARC expression and posed SPARC as a prognostic marker in hepatocellular carcinoma patients. This is novel in terms of the project plan without any wet laboratory experiments but only with the help of previous work and literature search with certain inclusion and exclusion criteria. The current manuscript is well written with some minor English grammar and typo mistakes. The pointwise comments are as follows:

1. Though the manuscript is well organized but is not easy to understand what is the goal/aim of this study.

**Response:** Thank you very much. The goal/aim of this study is to performed a meta-analysis to evaluate the relationship between the expression of secreted protein acidic and rich in cysteine (SPARC) and the prognosis of patients with hepatocellular carcinoma (HCC).

2. When there are many other proteins (PIMD 33312759, 34591393, 32439236, 28631558) studied in the HCC why SPARC has been chosen, and what is the rationale behind it?

**Response:** Thank you very much. SPARC is known to impact cancer growth in ambiguous ways, which either enhance or suppress cancer aggressiveness. (PMID: 32723563). Several studies reported that the expression of the SPARC could significantly predict a poor prognosis of gastric adenocarcinoma (PMID: 31127138). Meanwhile, SPARC expression was significantly upregulated in pancreatic adenocarcinoma and associated with poor prognosis (PMID: 33968201). Low levels of SPARC were associated with poor prognosis in human endometrial carcinoma (PMID: 33204109). However, the prognostic role of SPARC in liver hepatocellular carcinoma is still unclear (PMID: 34912848). Therefore, SPARC has been chosen in this study.

3. It would be better if the author had defined, what is “overall survival” and what is “disease-free survival”.

**Response:** Thank you for your valuable suggestions. The overall survival was calculated from the date of inclusion into the study until death or the date of the last follow-up (censored). Disease-free survival (DFS) was calculated from the date of surgery to the date of progression, recurrence, death, or final follow-up.

4. The entire manuscript is based on data mining and six paper has been selected which is a very small number.

**Response:** Thanks a lot. This research had some limitations due to included paper. However, our aim is to propose such a scientific hypothesis and conduct a meta-analysis based on existing research. Based on the conclusion of this paper, we believe that this is a relatively new scientific hypothesis, and this field deserves more attention in the future.

5. The total sample size based upon the 6 papers is 678, according to table 1 (120+89+79+130+60+200 = 678), why and how it is mentioned 698 in line 233 page 13 and line 256 page 14.

**Response:** Thank you very much for your kind comments. The wrong number “698” has been modified to “678”. Please see line 163 page 9, line 257 page 14, and line 279 page 15.

6. This manuscript is not a regular research article, many things in the manuscript have to be defined, like “method of group assignment,” (line 177 page 10) and “blinding in the outcome analysis” (line 178 page 10).

**Response:** Thank you very much for your helpful suggestions. Huang et al. used paired paraffin-embedded samples from patients with HCC who had undergone liver resection. Adjacent non-cancerous liver tissue samples were selected as the control group [35]. Liu el al. performed immunohistochemical assays in hepatic tissues collected from patients with HCC (study group) and healthy individuals (control group) [36]. Yang et al. selected patients with primary liver cancer who underwent surgery as the study group and tumor adjacent liver tissues as the control group [37]. Ju et al. selected patients with HCC matching the following criteria to form the study group: (1) pathologically diagnosed HCC; (2) no anticancer treatment or distant metastases before surgery; and (3) history of HCC resection, defined as macroscopically complete removal of the tumors. Meanwhile, a normal liver tissue pool from 10 healthy liver donors was assigned as the control group [32]. Zhang et al. selected HCC tissues as the study group and nontumorous tissues as the control group [33]. Darweesh et al. selected patients with HCC as the study group and healthy volunteers as the control group. Healthy paticipants were those who had normal liver enzyme levels and functions, negative results on viral hepatitis screening, and normal abdominal ultrasound scans [38]. Please see L185-201.

7. Instead of presenting in the tabular form, it would be better if all the 6 studies were described with a separate heading including the sample size, methodology used, and putative outcome.

**Response:** Thank you very much for your helpful suggestions. The patient characteristics are shown in Table 1. Huang et al. used quantitative polymerase chain reaction (qPCR) to determine SPARC levels in 120 samples. OS and DFS were recorded [35]. Liu et al. recorded OS and used immunohistochemistry (IHC) to determine SPARC levels in 89 samples [36]. Yang et al. used IHC to determine SPARC levels in 79 samples. OS and DFS were recorded [37]. Ju et al. used qPCR to determine SPARC levels in 130 samples. OS and relapse-free survival (RFS) were recorded [32]. Zhang et al. recorded OS and used methylmion specific polymerase chain reaction (MSP) to determine SPARC levels in 60 samples [33]. Darweesh et al. recorded OS and used qPCR to determine SPARC levels in 200 samples [38].We added lines describing the sample size, methodology used, and putative outcome of all the 6 studies. We believe information is demonstrated more clearly in the revised version. Please see L173-181.

8. Some sentences are incoherent, e.g. “The p53/p21Cip1/Waf1 pathway enhances the effects of radiotherapy and chemotherapy” what does this sentence mean, and how it is correlated with the study?

**Response:** Thank you very much. The confusing sentence has been removed in the revised version.

9. While talking about the limitations of the paper, on page no 14, it is mentioned “other types of biases” in line 260. It is better to describe the “other biases” or just delete this line.

**Response:** Thank you very much. The confusing sentence has been removed in the revised version.

10. What does TNM stand for in line 262, page 14?

**Response**: Thank you very much. TNM stands for Tumor Node Metastasis. It has been added in line 285, page 15.
